# Annotations

**Published:** 1895-06-22

**Authors:** 


### Annotations.

### Doctor or Schoolmaster.

We have been taken to task by the *Liverpool Post* for insisting that the science of physiology and the art of physic as applied to bodily and mental development shall have due recognition at our public and private schools. It has produced in our minds a condition of amazement that an intelligent newspaper writer should so signally fail to understand what seems to us to be so elementary and obvious. The farmer improves his cattle and his horses, his sheep and his pigs, and even his poultry and his dogs by methods strictly analogous to those insisted upon by the scientific physician: that is, he not only breeds rationally, but he feeds rationally, he places his growing stock in such conditions as to bring them up to the very highest standards of health, beauty of form, and practical efficiency, and he gives them just sufficient work to stimulate the highest development of their powers. In like manner, science is guiding the development of fruit, trees, plants of all kinds, roots, cereals, and the like, and that with the greatest possible advantage to the community. Does the writer in the *Post* then take up this position, that whilst science in her modern developments may be applied to horses and cattle, sheep and poultry, trees, roots and cereals, she is to have nothing whatever to say in the development of young human beings? If that be his position, what is his topical defence of it? Does he take his stand on the contention that all science is useless, or that boys are by nature so scientific that they do not need any external guidance; or that the schoolmaster, who knows little or nothing of science, is more competent to apply it in practice than the doctor, whose chief business in life is to reduce scientific principles to practice? The controversy seems to us to be one of the very first importance; and we would like very much to hear what the writer of the *Post's* article has further to say in justification of his startlingly retrograde position.

### The W. G. Grace Tribute.

The testimonial to Dr. W. G. Grace, which, started by the *Daily Telegraph*, has now assumed national proportions, ought, perhaps, to be called a "tribute" rather than a testimonial. A "tribute" of sincere admiration from all sorts of Englishmen in all parts of the world it most certainly is. Medical men, who consistently exalt physique and youthfulness in the middle-aged and the old, feel a peculiar satisfaction in the fact that the greatest athlete of the century, and the youngest middle-aged man, is one of themselves. The fact is a kind of accidental testimonial to our profession and to the merit of its teaching and work, as well as a tribute to Dr. Grace. Would Grace have been so youthful at forty-seven, and so perfect in eye, nerve, and muscle as he is at this moment, if he had not been animated by the spirit and intelligence of modern physic? We doubt it! The *Times*, inspired by the general enthusiasm, asks why W. G. was not remembered when the last Birthday honours were distributed? It is a natural and a generous question, and the answer probably is that the omission of his name from the list was a pure accident. But at any rate it is an accident which may yet be repaired. Of cricket itself, and of Grace's achievements therein, enough has been said, and will be said, in the lay press. For our part, though we are somewhat late in the day, in consequence of the crowding of our pages in the interests of Hospital Sunday last week, we profess our sincere admiration for the man who has raised to a pitch of unexampled popularity the noblest of English games. Our hope is that the national tribute may be as splendid as Dr. Grace's fame, and that the gracious Sovereign, who has so often interpreted the national feelings at their crowning moment, will, with the magic of her touch, give ideal completeness to the honours which an athletic nation so enthusiastically pours upon the head of the most distinguished of all the present representatives of modern athleticism.

### Frozen Milk.

We have become accustomed to frozen meat, and thaw our joint from the Antipodes, or other unknown sources, in calm indifference to its origin. But frozen milk is another matter. Our meat, at least, we cook, but milk is taken raw; and, in view of what we now know as to the possibility of its becoming a means of disseminating disease, it is impossible to hear without uneasiness that the introduction of frozen milk from abroad may at any moment assume enormous proportions. Not that we can pretend at present to be fit to pick the mote out of our brother's eye in regard to milk. The milk trade in England, from cow to kitchen, is at present often carried on under conditions which any man of sanitary instincts may well shudder to think of. But there are signs of improvement. Large dairy companies have set an example, and raised a standard to which other milk producers will have sooner or later to conform; the local sanitary authorities are exercising some modicum of control over the dairy farms within their districts; and the action of certain urban sanitary authorities in tabooing the milk from places which have been shown to be centres of infection has stimulated the farmers to greater carefulness. All these influences tend to raise the character of our home milk supplies, and it is hardly possible to believe that the report of the Royal Commission on Tuberculosis will not result in some attempt to improve it still further. What good purpose, however, will all this serve if the home markets are to be flooded with foreign milk, produced one knows not where, under conditions over which we cannot exercise the slightest control, brought frozen from the uttermost parts of the world, and of a character beaten down by universal competition to the lowest grade which will pass the very low standard accepted at Somerset House? Protection is in bad odour as a principle of international trade, but the British farmer would seem to have reason on his side in asking that, if his industry is to be subjected to interference, as it must be, for the sake of insuring for the people a good and wholesome milk supply, the foreign milk by which his own is beaten down in price shall also be subjected to equal supervision, and that either guarantees shall be demanded of its healthy origin, or it shall be sterilised before it is sold.

